# Angry and Afraid: Exploring the Impact of Mixed Emotional Reactions to Hate Crimes With LGBT+ and Muslim Communities

**DOI:** 10.1177/08862605241286455

**Published:** 2024-10-21

**Authors:** Jenny L. Paterson, Mark A. Walters, Rupert Brown, Diego Carrasco

**Affiliations:** 1 Northumbria University, Newcastle upon Tyne, UK; 2 University of Sussex, UK; 3 Pontificia Universidad Católica de Chile, Santiago, Chile

**Keywords:** hate crime, LGBT+, vicarious trauma, community violence

## Abstract

Hate crimes send messages of intolerance that can cause significant emotional and behavioral harm to entire identity groups. Previous research, based on intergroup emotions theory, has helped explain the psychological mechanisms that underpin the indirect effects of anti-LGBT+ hate crime, showing that incidents give rise to perceptions of threat among community members, which in turn elicit certain emotional reactions that trigger specific behavioral outcomes. This article provides two significant contributions to this developing knowledgebase. First, it provides an important replication of the theoretical model with another frequently targeted community: Muslim people. In addition, it offers the first quantitative analysis of how combinations of different emotions trigger discrete behavioral responses in the aftermath of hate crime, thereby providing much-needed nuance to the intergroup emotions theory model. Across two studies (Study 1: *N* = 589 LGBT+ participants; Study 2: *N* = 347 Muslim participants), we show that, for both LGBT+ and Muslim participants, indirect experiences of hate crimes are associated with greater perceptions of threat, which are then positively associated with anger, anxiety, and shame, that link to behavioral intentions: avoidance, pro-action, security behaviors, and retaliation. Latent class analyses further revealed that participants’ emotional reactions tend to cluster into four distinct profiles in both communities: people scored mid-range on all emotions, *or* high anger with low shame, *or* high anger with high anxiety, *or* low shame. These combinations had direct implications for intended behaviors across both groups: experiencing high anger with high anxiety was a cogent motivator of action. Most significantly, we provide new insights into *how* and *why* different emotions interact to predict both similar and divergent behaviors in the aftermath of hate crime incidents. Our findings yield important new knowledge that holds the potential of shaping both public policies and practices aimed at addressing the impacts of hate crimes.

A burgeoning body of research shows that hate crimes—which are crimes motivated by hostility or prejudice toward legally protected characteristics—have distinct impacts that are likely to *indirectly* affect entire communities of people (Holder, 2024; Lantz et al., 2024; Lantz & Kim, 2019; Paterson, Brown, & Walters, 2019a, 2019b; Pickles, 2021; Walters, Paterson, McDonnell, & Brown, 2020; Wenger et al., 2022). By specifically targeting victims’ group characteristics (e.g., religion and sexual orientation), hate crimes send a terroristic message to all those who share the victim’s identity or beliefs, resulting in what Iganski refers to as “waves of harm” (2001). Such messages can leave entire groups of people feeling threatened, angry, anxious, and even ashamed (Paterson, Brown, & Walters, 2019b). These group-based emotions, in turn, are thought to have distinct behavioral consequences, including avoiding certain locations, refraining from showing or expressing their identity traits, and, sometimes, forming the desire to retaliate (Bell & Perry, 2015; Lantz et al., 2024; Paterson, Brown, & Walters, 2019b; Perry & Alvi, 2012; Walters, Paterson, McDonnell, & Brown, 2020).

Although much is now known about the widespread impacts of hate crimes and the psychological mechanisms underpinning them (Paterson, Brown, & Walters, 2019a, 2019b), there remain significant gaps in the extant literature. First, the existing social psychological theoretical models explaining the indirect impacts of hate crime, to our knowledge, have only been developed and tested on one targeted group, the LGBT+ community (Paterson et al., 2018; Paterson, Brown, & Walters, 2019a, 2019b). As many other different characteristics and communities are targeted in hate crimes (e.g., race, religion, and disability), the generalizability of these models needs to be tested to both better reflect the range of communities that are targeted in hate crimes and to address the call for increased replicability in the social sciences (Nosek et al., 2022). Here, we provide a much-needed replication and examine the indirect impacts of hate crime, and its proposed mechanisms, with both LGBT+ (Study 1) and Muslim people (Study 2).

Furthermore, while the theoretical models propose hate crimes to elicit *discrete* emotions that motivate specific behaviors (e.g., Paterson, Brown, & Walters, 2019a), we acknowledge that in the aftermath of hate crime, people can feel a range of emotions *simultaneously* and so develop the model to examine how this *interplay of emotions* is likely to have differential and, as yet, unexplored impacts on behaviors. In doing so, our research provides new insights into the harms of hate crime across two commonly targeted communities. Given the high number of direct and indirect victims (Home Office, 2023), our findings offer a timely contribution to understanding the harms of hate crime.

## The Indirect Effects of Hate Crime: A Theoretical Model

Recent research has drawn on social identity theory; Tajfel & Turner, 1979) and intergroup emotions theory (IET; Smith, 1993) to develop theoretical models explaining the indirect impacts of hate crime (Paterson et al., 2018; Paterson, Brown, & Walters, 2019a, 2019b). At their core is the assertion that people form particularly strong attachments to others when they share similar identity-based traits (including ethnicity, religious beliefs, sexual orientation, and gender identities). The coalescence of groups around shared identity enables individuals to form a sense of belonging, purpose, and self-worth that facilitates their safe and secure navigation through the vicissitudes of life (Tajfel & Turner, 1979). The models suggest that when people categorize themselves as a member of a group (e.g., an LGBT+ person), they begin to view and react to the world, at least in part, in terms of the group’s collective identity.

Group identification has many implications for an individual’s sense of “self,” including the ways in which a person perceives and reacts to their lived environment. In the context of hate crimes, social identity theory and IET suggest that a direct attack on one ingroup member is likely to be felt and reacted to as an assault on *all* group members (e.g., Mackie & Smith, 2015). Furthermore, the models help specify the psychological mechanisms behind these group-based reactions and can be summarized in three steps: (a) indirect experiences of hate crime lead to group members feeling threatened, (b) this threat, in turn, elicits a range of emotional reactions, and (c) these emotions motivate specific behaviors. Below we explicate these links in further detail.

First, as hate crimes target both individual ingroup *members* and the overarching group *identity* (Paterson et al., 2024), these crimes pose both a realistic and symbolic threat to targeted groups (e.g., Stephan et al., 2015). For instance, group members fear that, due to their shared identity, *they* could be targeted next (realistic threat), while also becoming concerned about the resulting social marginalization of the *group* (symbolic threat). Indeed, previous studies have provided robust evidence of how indirect experiences of hate crime increase feelings of threat in both regards (Paterson, Brown, & Walters, 2019a, 2019b).

Second, according to IET, this appraisal of threat gives rise to specific emotional responses toward the crime (Mackie & Smith, 2015). Most commonly, *anger* is elicited because people are outraged by the injustice that arises when someone is attacked because of their identity trait; *anxiety* is increased because the attack highlights the relative weakness and vulnerability of the entire group; and, in some cases, *shame* is likely to increase in two distinct ways. The first is where the attack reinforces a dominant social lens through which an individual’s identity is being punished for being perceived as bad or immoral (Meyer, 2003). This can result in individuals internalizing the prejudices of others, for example, manifesting as “internalized racism” or “internalized homophobia” (e.g., Allen & Oleson, 1999). A second way in which group shame can be experienced is when the behavior of other ingroup members gives rise to a perception that the group image has been damaged (Paterson, Brown, & Walters, 2019b; Spears et al., 2011). Although a victim of hate crime has not directly “acted” to shame the group image, there is a connection between group members’ internalized feelings of prejudice (itself a result of shame) and a secondary form of group-based shame that is linked to the *perceived* behavior of direct victims via a mediating sentiment: *blame* (Bell & Perry, 2015). Blame can occur when group members experience heightened perceptions of symbolic threat and feelings of vulnerability due to the knowledge that group members have a heightened risk of hate crime victimization (Paterson, Brown, & Walters, 2019b). These perceptions and emotional responses can lead to a further perception that the direct victim must have done something to cause their own victimization, which Bell and Perry (2015) argue allows some group members to then distance themselves from the perceived risk of targeted victimization. The apportioning of blame to victims therefore gives rise to a sense that they have done something to damage the ingroup’s image, which in turn leads to feelings of shame among other group members.

Third, as these group-based emotions are motivated by different concerns (e.g., sense of injustice vs. perceived weakness), they can trigger different types of behavior (Smith, 1993; see also Cottrell & Neuberg, 2005 for a similar analysis). Anger, for example, is considered to motivate “’approach”’ behaviors and often leads to confrontation in an attempt to redress any power balance (Mackie & Smith, 2015). However, when elicited by a sense of injustice, this anger can also be a mobilizing force for social change, such as motivating individuals to promote social justice through collective action (Spears et al., 2011). These links have been evidenced to some extent in the hate crime literature, for instance, the anger elicited by indirectly experiencing anti-LGBT+ hate crimes has been associated with an increased desire to confront homophobia (Noelle, 2002), to educate others about identity “difference” (Perry & Alvi, 2012), and to join community-based groups that promote minority “rights” (Paterson, Brown, & Walters, 2019b). However, it has also been warned that feelings of anger *may* simultaneously give rise to retaliatory acts of hate-motivated violence, potentially fueling a pernicious cycle of hate-motivated crimes (Lantz et al., 2024; McDevitt et al., 2002). Although hypothesized as predictive of such behaviors, previous research has suggested it is not the emotion of anger *alone* that results in retaliatory intentions (Paterson, Brown, & Walters, 2019b), but that its combination with other emotions (e.g., empathy) may lead to a desire for retribution (Lickel et al., 2006).

Intergroup-based anxiety, meanwhile, is also a very common response to group-based threats and, because the anxiety reflects the perceived vulnerability of the group, this emotion motivates more avoidant behaviors (Stephan, 2014). Supporting this assertion, hate crime researchers have shown that hearing or reading about hate crimes can cause feelings of anxiety, which can prompt increased avoidance and cautiousness (Bell & Perry, 2015; Paterson, Brown, & Walters, 2019a, 2019b), being less open about their group-based identities (e.g., gay people being less likely to show same-sex intimacy in public, Noelle, 2002; Walters, Paterson, McDonnell, & Brown, 2020), and becoming more security conscious (e.g., improving house alarms; Paterson, Brown, & Walters, 2019b).

In addition to the well-documented behavioral impacts of anger and anxiety, shame is another important, yet overlooked, emotion that is often felt by victims of hate crime (e.g., Bell & Perry, 2015; Paterson, Brown, & Walters, 2019b). Beyond the field of hate crime studies, shame has been documented as manifesting behaviorally in myriad ways. Nathanson’s “compass of shame” predicts four categories of responses to a shame experience: withdrawal, avoidance, attack self, and attack other (Nathanson, 1994). Often unacknowledged by the person experiencing the emotion, Lewis (1971) grouped two distinct responses to shame that also aligns with Nathanson’s work. The first is referred to as “undifferentiated shame” and is characterized by a person experiencing negative assessments of themselves which results in withdrawal or silence. The second is labeled as “bypassed shame” and is associated with two other emotions anger and rage, which collectively can result in hostile forms of behavior.

This highly complex emotion can therefore lead to a variety of both approach *and* avoidance behaviors (Sheikh, 2014). For example, in the case of hate crime, to *protect* the group’s physical safety, (undifferentiated) shame may lead to avoidance to remove the (potential) victims from immediate harm and minimize their risk of (re)victimization (e.g., Iyer et al., 2007; Lickel et al., 2006). Alternatively, where (bypassed) shame gives rise to internalized feelings of humiliation, some group members may attempt to exert power and control over perpetrators through confrontation and even hostile retaliation (see also Ray et al., 2004).

## The Interplay of Emotions

Though understanding the impact of discrete emotions on behaviors has been a useful foundation in understanding the indirect impacts of hate crime, its utility may be somewhat limited in real-life situations (e.g., Spears et al., 2011). Both everyday observation and scientific research suggest that people feel more than one emotion simultaneously. Exemplifying this, Walters et al. (2020) interviewed 34 individuals identifying as Muslim or LGBT+ about their indirect experiences of hate crimes. They found that individuals commonly reported feeling anger, anxiety, *and* sadness in response to hearing about hate crimes. This mixture of emotions is understandable but complicates the somewhat simplistic IET predictions about behavioral consequences. For example, as anger is related to confrontation, but anxiety is associated with avoidance, what happens when individuals feel both anger and anxiety simultaneously? This is not just a theoretical question: understanding how the *combination* of these emotions can elicit different and competing behavioral motivations is crucial to providing a more granular understanding of the indirect effects of hate crime.

Acknowledging a lack of empirical research into mixed emotions, Spears et al. (2011) theorizes that experiencing anger and anxiety simultaneously may be particularly motivating and mobilizing, especially for marginalized groups. They suggest that, when people from marginalized groups feel anxious *and* they have nothing to lose, this anxiety can, paradoxically, lead to confrontation, especially when it is coupled with high levels of anger as this emotion tends to galvanize individuals.

Providing support for this hypothesis, Motro and Sullivan (2017) examined the roles of anxiety and anger in threatening interpersonal contexts. They found that anxiety-inducing contexts typically demotivated participants and led to avoidant behaviors. Crucially, however, when participants were also made to feel anger in these contexts, the combination of anxiety and anger was highly motivating and led to action (rather than inaction)—and these mixed emotions were more motivating than simply feeling anger alone. Extrapolating from the interpersonal domain, then, suggests that experiencing anger and anxiety together (vs. in isolation) may be particularly motivating and thus influential in predicting a wide range of behaviors in response to hate crimes (e.g., confrontation, retaliation, and pro-action).

Recognizing that individuals can and do experience anger and anxiety simultaneously is an important first step; however, it is just the beginning. As evidenced above, indirect victims of hate crime also report feeling shame (Paterson, Brown, & Walters, 2019b), and are thus likely to feel shame in combination with other emotions, which may further influence behaviors. Scheff (2019), for example, has posited that (bypassed) shame is likely to interact with anger to promote more hostile reactions, including violence. To our knowledge, however, there has yet to be an investigation into (a) what combinations of emotions people feel in response to hate crimes and (b) how experiencing these mixed emotions simultaneously influences behaviors. Such an investigation will produce a more complete understanding of the emotional interactions that motivate behavioral reactions to hate crimes.

## Present Research

As LGBT+ and Muslim people are common targets of hate crime in the United Kingdom (the site of this research; Home Office, 2023), we recruited participants identifying as LGBT+ (Study 1) and Muslim (Study 2) to examine the impacts of indirect experiences of hate crime. These groups were also chosen because of practicality issues (i.e., we believed we would be able to recruit a sufficient number of people from each group for the research), and to test the model’s replicability across groups that are targeted by different prejudices (i.e., against sexual orientation and/or gender identity vs. against a religion).

Across both studies, we hypothesized that having indirect experiences of hate crime would be positively associated with feelings of threat (H1a) which, in turn, would be positively associated with a range of emotions (anger, anxiety, shame, and H1b) that, subsequently, would be linked with behavioral intentions. We further predicted that anxiety would be positively associated with avoidant and security-based behavioral intentions, while anger would be positively associated with approach behavioral intentions, such as pro-action and confrontation. Shame, on the other hand, was expected to be related to both approach-orientated and avoidance-orientated behaviors (H1c).

Furthermore, we explored the emotional combinations participants reported after reading about a hate crime and how these mixtures of emotions were related to behavioral intentions. We hypothesized that the combination of feeling anxious and angry would be an especially motivating predictor of behavior resulting in stronger intentions to engage in pro-action, retaliation and security behaviors, and less avoidance (H2); however, due to the lack of previous empirical research into other emotion combinations, no further hypotheses for the interplay of emotions was proposed. Data and syntax from the studies can be found on the Open Science Framework: https://osf.io/aq8vu/?view_only=20e18570d79e4e2d8d96702eb3384820.^1^

## Study 1: Indirect Experiences of Anti-LGBT+ Hate Crimes

### Method

#### Participants

We aimed to recruit at least 300 participants for each study thereby exceeding the typical sample size used in path models (200: Klein, 2015). Moreover, as the populations under investigation are harder to reach than the general population, these sample sizes were pragmatic while allowing an adequate participant-parameter ratio of at least 5:1 to test for effects (Klein, 2015).

In total, 631 participants were recruited to the anonymous online survey via advertisements on social media and web links distributed by a variety of LGBT+ organizations in the United Kingdom. Respondents were offered entry into a prize draw for £100 in return for their participation. Of the 631 participants, 589 respondents who lived in Britain and indicated that they considered themselves to be LGBT+ were included in the final analysis. Participants were asked to indicate their gender by ticking which box(es) applied to them and/or providing their own gender identity. As a result, 354 selected male, 165 female, 19 trans female, 16 trans male, 16 trans, 5 genderqueer, 3 genderqueer trans, 1 genderqueer trans male, 1 genderqueer trans female, 1 nonbinary, 1 nonbinary female, 1 nonbinary trans, 1 nonbinary male, 1 intersex male, and 4 did not specify a gender. Ages ranged from 18 to 78 years (*M* = 34.67, *SD* = 13.38), and participants’ self-identification of their sexual orientation was as follows: gay (*n* = 330), lesbian (*n* = 91), bisexual (n = 81), queer (*n* = 32), pansexual (*n* = 17), straight (*n* = 15), asexual (*n* = 8), homosexual (*n* = 7), and eight people did not specify their sexual orientation. The vast majority of participants identified as White (*n* = 494), 26 people indicated they were from mixed/multiple ethnic backgrounds, 13 people identified as Asian, 5 people identified as Black, and 51 participants did not specify their ethnicity.

#### Measures

All measures, unless otherwise stated, were measured on a 7-point Likert scale. All scales were factor analyzed and any items with poor loadings (<.40) were dropped prior to analysis. Affected items are specified below.

To ensure a range of experiences of hate-motivated acts were captured, we created a scale that asked participants about their experiences of both hate *crimes* (criminal offenses perceived to be motivated by anti-LGBT+ prejudice) and hate *incidents* (noncrime incidents perceived to be motivated by anti-LGBT+ prejudice) and included five broad categories: verbal abuse, online abuse/harassment, vandalism, physical assault without a weapon, and physical assault with a weapon.

For *direct experiences of hate crimes*, participants were instructed to report all the times, in the past 3 years, that they themselves had been a victim of the five incidents when “the attacker(s) were (partly) motivated by a prejudice against LGBT+ people.” Categorized response options were initially used (e.g., 0 times and 1–3 times), however, since the data were skewed, we dichotomized the responses to reflect those who had been a victim of the crime and those who had not. We then created a mean score with these five dichotomous values (α = .51).

*Indirect experiences of hate crimes* were similarly assessed. Participants reported the number of people they knew who had been victims of the five types of anti-LGBT+ hate crimes in the past 3 years. Response options were “Nobody,” “1–3 people,” “4–6 people,” “7–9 people,” “10–12 people,” “13–15 people,” and “16 people or more” but, similar to the previous scale, we dichotomized the responses and created a mean score with these five dichotomous values (α = .68).

*Perceived threat* was measured by nine items drawn from two separate but conceptually similar scales. Four items were amended from Cottrell and Neuberg (2005) and used the question stem, “I believe anti-LGBT+ hate crimes and hate incidents pose a real threat to. . .” and included items such as “The rights of LGBT+ people in the UK.” The other five items tapped into personal vulnerability, for example, “Being LGBT+ makes me feel vulnerable to anti-LGBT+ hate crimes and incidents” (α = .89).

We created three scales to assess emotional reactions toward hate crimes and used the following prompt: “Please imagine that you find out that an LGBT+ person, who you did not personally know, was physically assaulted in an anti-LGBT+ hate crime in the town where you live. To what extent would you feel the following emotions. . ..” *Anxiety* was measured by “Anxious,” “Afraid,” and “Alarmed,” α = .87. *Shame* was measured with three items: “Embarrassed,” “Ashamed,” “Guilty,” α = .79. *Anger* was measured by “Angry,” “Annoyed,” “Outraged,” and “Appalled,” α = .76. We had initially included “Disgust” and “Revolted” to measure anger but, on reflection, these items are theoretically and practically distinct from anger (Mackie & Smith, 2015) and were so dropped from the scale.

To measure *Avoidance, Pro-Action, Security*, and *Retaliatory* behavioral intentions, participants were instructed, “Still imagining that you have found out about an LGBT+ person, who you did not personally know, was physically assaulted in an anti-LGBT+ hate crime in the town where you live, to what extent would you do the following. . .?.”

Using scales that we created, *Avoidance* was measured by seven items, including “I would go out less often” (α = .86). An eighth, reversed-scored, item of “I wouldn’t change my daily activities” did not load well and so was dropped from the scale. *Pro-action*, which represents a form of collective action, was measured by three items including “I would join and/or increase my participation in anti-hate crime groups” (α = .90). *Security* was measured with two items, “I would enroll in a self-defense class,” and “I would improve the security of my home and my personal belongings” (*r* = .51). *Retaliation* was measured with two items: “If I could, I would try to get my own back on the offenders in some way,” and “If I could, I would help others to get their own back on the offenders in some way.” As these two items had highly skewed distributions (many zero responses), we treated retaliation as dichotomous where 0 = explicitly disagreed with any retaliation on both items (i.e., indicated 3 or less on *both* items) versus 1 = possible retaliation on either or both items (i.e., indicated 4 or more on *either* items; *r_tet_* = .99).

Participants also reported the strength of their *LGBT+ identity* as measured by four statements adapted from Leach et al. (2008), including “I feel good about being an LGBT+ individual,” α = .83. A fifth, reversed-scored item (“I dislike being LGBT”) did not load well and was dropped from the scale.

## Results

The data revealed that 92% of respondents had been either a direct and/or an indirect victim of hate crime/incident in the 3 years prior to the survey. While previous research has found around 70% of LGBT+ respondents knew of a victim of a hate crime and around 35% had been a direct victim (Paterson, Brown, & Walters, 2019a), the current study also measured hate *incidents*—acts which may not meet the threshold for a crime yet are still motivated by hate—which may account for the slightly higher number in this research. Means and zero-order correlations between the variables are presented in Table 1. The most endorsed emotion was anger, followed by anxiety, while shame was somewhat less in evidence but still non-negligible. Pro-action was the most endorsed intended behavior followed by avoidance and increasing security, while over a quarter of the sample endorsed some form of retaliation. Consistent with expectations, experiences of hate crimes mostly correlated with the threat, emotional, and behavioral intention variables in the expected directions (note: we used a relatively conservative level of significance (p < .01) in view of the large number of correlations reported). Note also the relatively high mean level of LGBT+ identification (*M* = 5.37); this supports our general assumption that an intergroup emotions analysis is appropriate, predicated as it is on some minimal level of identification with the targeted group.

**Table 1.** Study 1: Means and Zero-Order Correlations Among Variables for LGBT+ Sample.

Variables	1	2	3	4	5	6	7	8	9	10	11
1. Indirect	—	.48**	.31**	.06	.14*	.11*	.22**	.12**	.15**	.02	.05
2. Direct		—	.36**	.11*	.22**	.12**	.19*	.21**	.24**	.05	−.08
3. Threat			—	.30**	.58**	.19**	.31**	.36**	.57**	.09	.01
4. Anger				—	.42**	.17**	.29**	.18**	.16**	.09	.19**
5. Anxiety					—	.35**	.33**	.38**	.63**	.04	.03
6. Shame						—	.27**	.27**	.31**	.09	−.08
7. Pro-action							—	.46**	.27**	.14**	.15**
8. Security								—	.44**	.15**	.03
9. Avoidance									—	.05	−.17**
10. Retaliation										—	−.00
11. Identification											—
Mean (*SD*)	0.48 (0.30)	0.24 (0.22)	4.73 (1.25)	6.12 (1.01)	5.05 (1.55)	2.53 (1.63)	4.08 (1.59)	3.00 (1.51)	3.78 (1.38)	0.27 (0.44)	5.37 (1.19)

* *p* < .01. **p ≤ .001.

### Path Model

**Figure 1.**
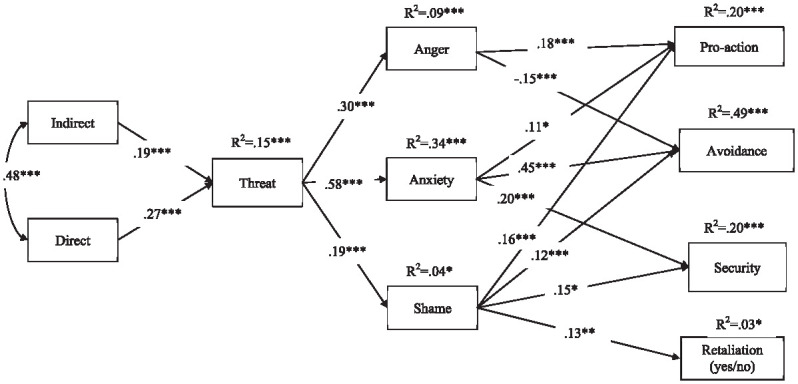
Study 1: Path model for LGBT+ sample. Note. All paths are standardized. χ^2^ (6) = 6.01; *p* = .42; SF = 1.14; CFI = 1.00; RMSEA = .002 (95% CI [0.000, 0.05]); SRMR = .01. *p < .05. **p < .01. ***p < .001.

We next tested the hypothesized path model using Mplus version 6 (Muthén & Muthén, 2011). In the model (Figure 1), the emotional mediating variables (anger, anxiety, and shame) were set at the same level and allowed to covary. The behavioral intention measures (avoidance, pro-action, security, and retaliation) were set at the same level and allowed to covary. In addition to the paths shown in Figure 1, we also predicted direct paths from the exogenous variables (indirect and direct experiences) and threat variables to the behavioral intentions. The data fit the model well, χ^2^ = 6.01, *p* = .42 (*df* = 6; scaling factor = 1.14), CFI = 1.00, RMSEA = .002 (95% CI [0.000, 0.05]), SRMR = .01 (Hu & Bentler, 1999).

As predicted by H1a, Figure 1 shows that both direct and indirect victimization experiences were associated with appraising hate crimes as a group-based threat. Supporting H1b, this increase in threat was, in turn, associated with increases in anger, anxiety, and shame. The strength of the association between threat and emotions varied considerably, from β = .58 (anxiety) to β = .19 (shame), as did the amount of variance accounted for in the emotions (from 4% to 34%).

As predicted by H1c, the next stage of the model revealed that these emotions were differentially linked to specific behavioral intentions. Anger, as an approach-orientated emotion, was linked to pro-action positively and negatively to avoidance. Anxiety was also positively associated with pro-action but, in contrast to anger and consistent with its defensive orientation, was also linked to increases in avoidant and security-related behaviors. Despite the fact that mean levels were relatively low, shame was positively associated with all four behavioral intentions. It was the *only* emotion to be reliably associated with retaliation.

### Mixed Emotions: Latent Class Analysis and Comparisons

Latent class analysis (LCA), which is a clustering technique that estimates categories based on the response patterns on a collection of variables (Vermunt & Magidson, 2002), was used to categorize participants into groups based on their emotional responses. After testing the fit of six different models with varying numbers of classes (1–6; see Table 2 for model fit indices), we selected four classes as they presented the lowest Bayesian information criteria (BIC; 20,119.85) and presented good classification precision (E_k_ = .89; Classification Error = .04; Nylund et al., 2007). These four classes are characterized by the proportion of participants who reported high extreme scores on anger and anxiety (i.e., 7s on the 1–7 scale), and low extreme scores on shame (i.e., 1 on the 1–7 scale) and are as follows: *Class 1: Consistent emotion* (33.3% of participants generally scored mid-range on all emotions); *Class 2: High anger and Low shame* (26.3% of participants reported high anger and low shame scores); *Class 3: High anger and High anxiety* (26.3% of participants reported high anger together with high anxiety); and *Class 4: Low shame* (14.1% of participants reported low shame).

**Table 2.** Study 1: Model Fit Indices for the Tested Latent Class Models.

Classes	BIC	AIC	Param.	*L* ^2^	% Change *L*^2^	Class. Error	E_k_
1	21,463.36	21,148.12	72	13,683.62			
2	20,321.35	19,686.48	145	12,075.98	0.12	0.03	0.89
3	20,129.64	19,175.15	218	11,418.65	0.05	0.04	0.89
4	20,119.85	18,845.73	291	10,943.23	0.04	0.04	0.91
5	20,336.10	18,742.35	364	10,693.85	0.02	0.05	0.91
6	20,557.13	18,643.76	437	10,449.26	0.02	0.06	0.92

*Note.* BIC = Bayesian information criteria; AIC = Akaike’s information criterion; Param. = number of parameters estimated in the model; *L*^2^ = likelihood ratio Chi-square statistic; % change *L*^2^ = percentage of change of *L*^2^ between k–1 and k class model; Class. Error = classiﬁcation error; E_k_ = relative entropy.

Having classified participants into the four classes according to their predominant emotional responses, we next examined which classes were most prone to behavioral intentions, with the expectation that *Class 3: High anger and high anxiety* would report higher intentions for pro-action, retaliation and security behaviors, and lower intentions to avoid (H2). Because variances were not equal across groups and measures, we used multiple group median test comparison (Conover, 1999) for *avoidance*, *pro-action*, and *security*. This test provides a Chi-square test for a null hypothesis where there is an equal number of observations above and below the median for all the observations (an omnibus test) and then provides a test for the median of each pair of groups using a similar procedure (de Mendiburu, 2023). We used a Bonferroni correction for all comparisons within each outcome (α/4 = .05/4). For *retaliation*, we used a pairwise proportion test conditional to latent class and used a Chi-square as an omnibus test and compared all pairs of proportions using a Bonferroni correction as well.

As shown in Table 3, participants who were characterized as having *high anger and high anxiety (Class 3)* were more likely to engage in all behaviors compared to the other classes of participants, including avoidance contrary to hypotheses. In addition, participants characterized as having *high anger and low shame (Class 2)* were also more likely to want retaliation in comparison to the rest of the groups.

**Table 3.** Study 1: Multiple Group Median Test of Behavioral Intentions Across the Mixed Emotions Latent Classes (LGBT+ Sample).

Variable	Overall χ^2^ Statistic (*df*)	Class 1: Consistent Emotion	Class 2: High Anger, Low Shame	Class 3: High Anger, High Anxiety	Class 4: Low Shame
Avoidance^d^	98.39(3)***	4.00_b_	3.14_c_	4.71_a_	2.71_c_
Pro-action^d^	35.87(3)***	4.00_b_	4.33_b_	5.00_a_	3.00_c_
Security^d^	35.54(3)***	3.00_b_	2.50_b_	4.00_a_	2.00_c_
Retaliation^e^	16.70(3)***	0.22_b_	0.35_a_	0.32_a_	0.14_b_

*Note.* χ^2^ is reported with degrees of freedom in parenthesis.

Rows with different subscripts (a, b, and c) indicate significant differences at *p* < .05/4 (Bonferroni corrected).

d Multiple median comparisons.

e Multiple proportion comparison.

* *p* < .05. **p  < .01. ***p  < .001.

## Study 2: Indirect Experiences of Islamophobic Hate Crimes

### Method

#### Participants

Participants were recruited via online advertisements on social media and Muslim organizations in the UK in return for entry into a prize draw for £100. Out of the initial 406 participants recruited, only those who indicated that they were Muslim and lived in the UK were included in the analyses (*n* = 347; 151 males, 195 females, 1 unspecified; *M*_age_ = 33.37, *SD* = 12.52, range 17–75). The majority of participants identified as Asian (*n* = 204), 51 identified as White, 40 as Arab, 18 as multiple or mixed ethnicity, 13 as Black, and 21 specified other or preferred not to answer. Most participants were British (*n* = 227), with the remainder from a variety of countries in South Asia, Africa, and the Middle East (e.g., Pakistan, Bangladesh, Saudi Arabia, India, and Nigeria).

#### Measures

Measures were similar to those used in Study 1, with some minor wording differences to reflect the nature of the new victimized group (e.g., referring to Islamophobic hate crimes rather than anti-LGBT+ hate crimes). To maintain consistency across the studies, the items that were dropped in Study 1 were also dropped in this Study. All scales were reliable: *Direct experiences of hate crimes* α = .65, *Indirect experiences* α = .79, *Perceived threat* α = .89, *Anger* α = .90, *Anxiety* α = .86; *Shame*, α = .82, *Pro-Action* α = .87, *Security* r = .54, *Avoidance* α = .79, *Retaliation r_tet_* = .99, *Muslim identification* α = .94.

## Results

Illustrating the pervasiveness of Islamophobic hate crimes and incidents, 88% of participants had been either a direct and/or indirect victim of an Islamophobic hate crime/incident in the 3 years preceding the study. Means and zero-order correlations between the variables are presented in Table 4. As in Study 1, anger and anxiety were the most endorsed emotions, followed by shame. The behavioral intentions of pro-action and security were endorsed, with avoidance rather less so. Some form of retaliation was endorsed by 34% of participants. Experiences of indirect and direct hate crimes significantly correlated with most variables in the expected directions. As in Study 1, the mean level of identification with the ingroup was quite high (*M* = 5.81) further supporting the IET approach.

**Table 4.** Study 2: Scale Means and Zero-Order Correlations Among Variables for Muslim Sample.

Variables	1	2	3	4	5	6	7	8	9	10	11
1. Indirect	—	.61**	.50**	.39**	.36**	.07	.31**	.28**	.31**	.06	.21**
2. Direct		—	.39**	.23**	.21**	.06	.24**	.29**	.30**	.15*	.08
3. Threat			—	.50**	.60**	.10	.39**	.38**	.49**	.00	.33**
4. Anger				—	.54**	.19**	.48**	.35**	.30**	.03	.46**
5. Anxiety					—	.22**	.38**	.41**	.66**	−.04	.33**
6. Shame						—	.19**	.21**	.23**	.20**	−.01
7. Pro-action							—	.50**	.31**	.08	.33**
8. Security								—	.44**	.17**	.21**
9. Avoidance									—	.04	.14*
10. Retaliation										—	−.02
11. Identification											—
Mean (*SD*)	0.53 (0.35)	0.30 (0.26)	4.77 (1.54)	5.94 (1.51)	5.20 (1.80)	2.57 (1.84)	4.66 (1.71)	4.03 (1.76)	3.82 (1.44)	0.34 (0.48)	5.81 (1.58)

* *p* < .01, **p ≤ .001.

### Path Model

**Figure 2.**
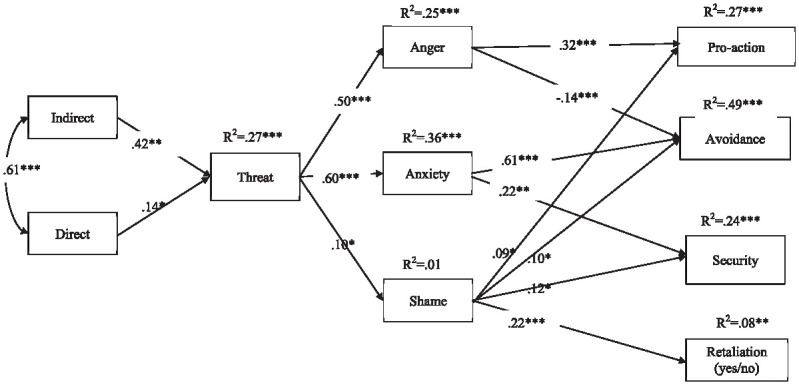
Study 2: Path model for Muslim sample. *Note.* All paths are standardized. χ^2^(6) = 14.40; *p* < .001; SF = 1.12; CFI = .99; RMSEA = .06 (95% CI [0.02, 0.11]); SRMR = .02. *p < .05. **p < .01. ***p < .001.

We again tested the hypothesized model using Mplus (Muthén & Muthén, 2011). The model showed acceptable fit, χ^2^ = 14.40, p = .03 (df = 6, scaling factor = 1.12), CFI = .99, RMSEA = .06 (95% CI [0.02, 0.11]), SRMR =0.02 (Hu & Bentler, 1999). The significant paths of the model are consistent with the LGBT+ study and hypotheses (Figure 2). Frequency of indirect victimization was positively associated with threat (H1a), and threat was positively associated with the three emotional reactions (anger, anxiety, and shame: H1b). Shame was again positively correlated with all the behavioral intentions, anger was linked positively to pro-action and negatively to avoidance, while anxiety was associated with avoidance and security (H1c). The only link not to be replicated in this sample was the significant positive association between anxiety and pro-action found in the LGBT+ sample.

### Mixed Emotions: LCA and Comparisons

Repeating the same strategy as in Study 1, we used LCA to classify participants into four groups according to their responses across all emotions (see Table 5 for model fit indices). Although the three-class solution presented a smaller BIC for this sample alone (BIC = 11,239.85, *E_k_* = .92, Classification Error = 0.03), a model with four classes (BIC = 11,365.12, *E_k_* = .87, Classification Error = 0.02) was preferred. The four latent class solution is preferable because it is consistent with the previous classification without increasing the overall classification error of the fitted model. Moreover, we believe the low prevalence of one latent class in the Muslim group (Class 4: Low shame) is working as an artifact, producing lower BIC values for the three-class solution for the Muslim group alone (Morgan, 2015). Thus, we fit a series of multigroup LCA of 2 to 6 classes for the LGBT and Muslim samples simultaneously (Table 6). We compared all the fitted models, which again favored the four-class solution (BIC = 30,647.83, *E_k_* = .91, Classification Error = 0.05). Thus, we used the four latent class solutions fitted onto the Muslim sample for the following comparisons *Class 1: Consistent emotion* (20.5% of participants); *Class 2: High anger and low shame* (39.8% of participants); *Class 3: High anger and high anxiety* (32.0%); and *Class 4: Low shame* (7.7% of participants).

**Table 5.** Study 2: Model Fit Indices for the Tested Latent Class Models.

Classes	BIC	AIC	Param.	*L* ^2^	% Change *L*^2^	Class. Error	*E_k_*
1	12,288.26	12,011.11	72	8,009.43			
2	11,560.88	11,002.73	145	6,865.62	0.14	0.02	0.93
3	11,239.85	10,400.70	218	6,129.43	0.11	0.03	0.94
4	11,365.12	10,244.96	291	5,827.96	0.05	0.02	0.96
5	11,531.58	10,130.43	364	5,573.67	0.04	0.02	0.97
6	11,740.97	10,058.81	437	5,358.96	0.04	0.02	0.97

*Note.* BIC = Bayesian information criteria; AIC = Akaike’s information criterion; Param. = number of parameters estimated in the model; *L*^2^ = likelihood ratio Chi-square statistic; % Change *L*^2^ = percentage of change of *L*^2^ between k–1 and k class model; Class. Error = Classiﬁcation error; *E_k_* = relative entropy.

**Table 6.** Study 2: Model Fit Indices for the Tested Multigroup Latent Class Models.

Classes	BIC	AIC	Param.	*L* ^2^	% Change *L*^2^	Class. Error	*E_k_*
2	31,507.51	30,800.64	146	19,485.68		0.03	0.89
3	30,787.81	29,722.66	220	18,259.71	0.06	0.04	0.92
4	30,647.83	29,224.40	294	17,613.44	0.04	0.05	0.91
5	30,667.16	28,885.45	368	17,126.49	0.03	0.06	0.91
6	30,739.69	28,599.70	442	16,692.75	0.03	0.07	0.90

*Note.* BIC = Bayesian information criteria; AIC = Akaike’s information criterion; Param. = number of parameters estimated in the model; *L*^2^ = likelihood ratio Chi-square statistic; % Change *L*^2^ = percentage of change of *L*^2^ between k–1 and k class model; Class. Error = Classiﬁcation error; *E_k_* = relative entropy.

We again used the multiple group median test (Conover, 1999) to assess any differences between the groups on the behavioral intentions of *avoidance*, *pro-action*, and *security*, and a pairwise multiple proportion test for *retaliation*. We used a Bonferroni correction for all pairwise comparisons among expected latent classes. Again, providing support for H2, Table 7 shows that participants characterized as *high anger and high anxiety (Class 3)* were more likely to engage in pro-action and security-related behaviors than those reporting *consistent emotion (Class 1)* and *low shame (Class 4)*, though the differences were not significant between participants reporting *high anger and low shame (Class 2)*. Replicating Study 1, the exploratory analyses also revealed that those who reported *high anger and low shame* (*Class 2*) were more likely to engage in all behaviors compared to those categorized as having only *low shame* (Class 4), though the difference in retaliation was not significant (χ^2^ = 4.10, p = .25).

**Table 7.** Study 2: Multiple Group Median Test of Behavioral Intentions Across the Mixed Emotions Latent Classes (Muslim Sample).

Variable	Overall χ^2^ Statistic (*df*)	Class 1: Consistent Emotion	Class 2: High Anger, Low Shame	Class 3: High Anger, High Anxiety	Class 4: Low Shame
Avoidance^d^	26.94(3)***	4.00_a_	3.83_a_	4.00_a_	1.67_b_
Pro-action^d^	36.89(3)***	4.67_b_	5.00_a_	5.67_a_	1.50_c_
Security^d^	22.09(3)***	4.00_b_	4.25_ab_	4.50_a_	1.00_c_
Retaliation^e^	4.10(3)	0.35_a_	0.31_a_	0.43_a_	0.25_a_

*Note.* χ^2^ is reported with degrees of freedom in parenthesis.

Rows with different subscripts (a, b, and c) indicate significant differences at *p* < .05/4 (Bonferroni corrected).

d Multiple median comparisons.

e Multiple proportion comparison.

* *p* < .05, **p < .01, ***p < .001.

## Discussion

Utilizing data from two commonly targeted communities, our investigation provides a much-needed replication (Nosek et al., 2022) and more comprehensive understanding of the indirect impacts of hate crime. Expanding upon previous research primarily focused on anti-LGBT+ hate crimes (e.g., Paterson et al., 2018; Paterson, Brown, & Walters, 2019a, 2019b), we find clear and consistent support for the proposed model in both LGBT+ and Muslim samples. Consistent with predictions, people who have indirect experience of hate crimes view such crimes as threatening intergroup contexts (H1a), which predictably elicit group-based anger, anxiety, and shame (H1b). These emotions, in turn, have specific behavioral consequences (H1c): anxiety leads to avoidance and security-related behaviors; anger leads to less avoidance and more pro-action; while shame—although not felt as strongly as the other emotions—significantly predicts all behaviors, including uniquely associating with retaliation. As only one association (anxiety to pro-action) did not replicate across both samples, the striking similarity between the two studies provides evidence for the generalizability of previous findings that indirect experiences of hate crimes have significant and consistent types of impacts on victims across different targeted communities.

The consistent links between emotions and behavioral intentions in both communities are particularly notable. We provide further evidence of the more pronounced (and more researched) emotions of anger and anxiety, along with their respective approach and avoidant outcomes (e.g., Paterson, Brown, & Walters, 2019a). Moreover, our research provides empirical support for the claims made by leading theorists on the importance of shame (e.g., Nathanson, 1994; Scheff, 2019), showing that it predicts both avoidant and retaliatory behavioral intentions. Adding to this, we offer new insights into responses by showing that shame may also serve as a conduit to pro-action that serves to mobilize group identity. In this regard, the studies support the contention that shame really is the “master emotion” (Scheff, 1997).

Adding important nuance to the findings, we provide the first quantitative evidence showing how a mixture of emotions in response to hate crime is associated with different behavioral intentions. Supporting previous theorizing (Spears et al., 2011), both studies revealed that participants who reported heightened levels of anger *together* with heightened levels of anxiety were significantly more likely to intend to respond to hate crimes in proactive and security-conscious ways than other participants. The combination of anger and anxiety in the LGBT+ sample was also related to an increased intent to engage in retaliation, though, counter to hypotheses, these participants were also more likely to intend to engage in avoidance.

In addition to this galvanizing effect of anger on anxiety, our exploratory analyses revealed that anger also stimulates those who feel little shame. Compared to those who were characterized as feeling low amounts of shame with no other extreme scores (*low shame* groups), participants who reported low shame together with extreme anger were significantly more likely to engage in all behaviors (though the difference in retaliation for the Muslim sample was non-significant). Together, the findings clearly show that feeling extreme anger has a mobilizing effect on behavioral intentions (Mackie & Smith, 2015), however, the exact influence on behaviors is likely to be determined by the combination of emotions felt alongside anger (e.g., high anxiety or low shame). Indeed, according to Scheff (2019) experiencing anger in conjunction with higher levels of shame is likely to heighten both emotions even further, ultimately escalating into violence. Although there were not enough participants reporting both high anger and high shame to constitute a distinct class in our analyses, considering our path models show that shame is independently and positively correlated with retaliation, and the LCAs show that the addition of high anger strengthens behavioral intentions, it is conceivable that if and when people experience both high anger and high shame, they may be more motivated to retaliate. Such theorizing exemplifies the necessity of considering multiple and mixed emotions to fully understand the true impacts of hate crimes.

### Limitations

While the research clearly adds to the literature, it is not without its limitations. First, the correlational design precludes causal inferences, and it could be that experiencing *any* type of crime is sufficient to elicit emotional reactions and behavioral responses. However, previous experimental data shows that hate crimes are more likely to impact victims emotionally compared to non-hate-motivated crimes, specifically because of the hate element of the crime (Study 2, Paterson, Brown, & Walters, 2019a). Second, we also acknowledge that the (hypothetical) method used to elicit emotions and behavioral intentions was not ideal since it was less direct than asking participants to respond to their own hate crime experiences (whether direct or indirect). However, this approach allowed us to compare the emotions and behavioral intentions elicited from the *same* standardized situation, thereby reducing the influence of other variables, including factors such as closeness to the victim (e.g., friend and relative) and severity of the hate crime, among many other possible confounding variables. Our measures could also be improved upon. Although the scales we developed for this research allowed greater flexibility and were generally reliable, future research could further validate these items. In addition, alternative standardized measures could expand the model further, for example, measures assessing outgroup prejudice could assess to what extent hate crime experiences fuel the “cycle of hate” (Lantz et al., 2024),

Although we assume that the psychological processes underpinning the reactions to hate crime will be the same for any group targeted—and our replication in Study 2 provides initial support for this—further research should test the hypotheses with groups targeted because of other prejudices (e.g., racism and ablism) and in different countries and contexts with different group histories and conflicts. Similarly, as the LGBT+ and Muslim communities consist of millions of diverse people with a range of different identities, which could further impact people’s experiences and responses, future research would be well placed to examine how different identities (e.g., social class, nationality, age, education level, and sex), contexts (group histories), and important subgroup identities (e.g., lesbian vs. gay; Sunni vs. Shia), as well as the intersectionality of these factors, may lead to different experiences of hate crime and, therefore, different responses.

### Policy Implications

The data clearly show that Muslim and LGBT+ individuals continue to face substantial forms of violent prejudice in the United Kingdom. Hate crime is a highly common experience for these individuals, both direct and indirect, which has substantial negative impacts on entire groups’ capacity to participate fully and equally in society (see Walters, 2022). As indirect victimization is associated with different behavioral intentions (pro-action and avoidance) mediated by various emotional reactions (anger, anxiety, and shame), those working to support victims can use these findings to tailor interventions that carefully consider how and when mixed emotions result in divergent behaviors. In particular, both state and community-based organizations should consider measures that prudently facilitate and manage the mobilizing emotions of anger and anxiety. Simultaneously, they ought to explore ways of mitigating against the negative effects of anxiety and shame, reactions that result in avoidant behavior (social harms), while also identifying the circumstances where incidents give rise to a desire for violent retaliation.

We do not underestimate the complexity of such a task. We hope, though, that our findings offer useful insights to those tasked with reducing the harms of hate crime; allowing both policymakers and justice professionals to better comprehend the various dynamics underpinning why and how communities respond to the increasing phenomenon of hate crime.

## Notes

1. The results presented here form part of a larger research project that collected data from the same participants on a range of other constructs related to hate crime. This included participants’ attitudes toward the criminal justice system and restorative justice which have resulted in three publications (Paterson, Walters, & Brown, 2019; Walters et al., 2017; Walters, Paterson, & Brown, 2020). However, the aims of this paper are distinct, and the path models and the mixed emotions analyses have not previously been published.
